# Making Sense of Intracellular Nucleic Acid Sensing in Type I Interferon Activation in Sjögren’s Syndrome

**DOI:** 10.3390/jcm10030532

**Published:** 2021-02-02

**Authors:** Erika Huijser, Marjan A. Versnel

**Affiliations:** Department of Immunology, Erasmus MC, University Medical Center Rotterdam, 3015 GD Rotterdam, The Netherlands; e.huijser@erasmusmc.nl

**Keywords:** Sjögren’s syndrome, autoimmune, cytosolic pattern recognition receptors, nucleic acid sensors, interferon

## Abstract

Primary Sjögren’s syndrome (pSS) is a systemic autoimmune rheumatic disease characterized by dryness of the eyes and mucous membranes, which can be accompanied by various extraglandular autoimmune manifestations. The majority of patients exhibit persistent systemic activation of the type I interferon (IFN) system, a feature that is shared with other systemic autoimmune diseases. Type I IFNs are integral to anti-viral immunity and are produced in response to stimulation of pattern recognition receptors, among which nucleic acid (NA) receptors. Dysregulated detection of endogenous NAs has been widely implicated in the pathogenesis of systemic autoimmune diseases. Stimulation of endosomal Toll-like receptors by NA-containing immune complexes are considered to contribute to the systemic type I IFN activation. Accumulating evidence suggest additional roles for cytosolic NA-sensing pathways in the pathogenesis of systemic autoimmune rheumatic diseases. In this review, we will provide an overview of the functions and signaling of intracellular RNA- and DNA-sensing receptors and summarize the evidence for a potential role of these receptors in the pathogenesis of pSS and the sustained systemic type I IFN activation.

## 1. Introduction

The immunogenicity of nucleic acids (NAs) and their shaping of the immune response has been recognized for many decades [1,2]. The discovery of pattern recognition receptors (PRRs) has provided a molecular mechanism for these observations [3]. Although at first NA-sensing receptors were thought to mainly sense pathogen-derived NAs, currently they are widely recognized to be able to sense self-NAs as well [4,5,6]. Upon activation, NA-sensing receptors induce the production of pro-inflammatory cytokines and type I interferons (IFNs) [4,7,8].

Type I IFNs are highly potent cytokines with direct anti-viral effects and a wide range of immunomodulatory functions [9,10]. Inappropriate amplitude and timing of type I IFN responses has detrimental effects on defense against pathogens and host tissue integrity [11]. Persistent systemic type I IFN activation occurs in primary Sjögren’s syndrome (pSS) and other systemic autoimmune diseases and is considered to contribute to the ongoing loop of inflammation [12].

Lymphocytic infiltrates in exocrine glands and symptoms of dryness of eyes and mouth are typical features of pSS [13]. The majority of patients with pSS and related systemic autoimmune diseases have anti-nuclear antibodies, reflecting the exposure of nuclear components to the immune system. Internalization of immune complex-bound NAs and activation of endosomal PRRs is thought to represent a major type I IFN-stimulating mechanism in these patients [14,15,16]. Recent identification of additional IFN-inducing NAs and detection of activated downstream sensing pathways suggested their potential contribution to IFN activation in systemic autoimmune diseases [17,18,19,20,21,22,23].

Here we will outline the signaling and regulation of intracellular RNA- and DNA-sensing pathways and discuss the current knowledge on the role of these pathways in type I IFN activation in pSS.

## 2. Type I IFN Signaling in Primary Sjögren’s Syndrome

### 2.1. IFN Cytokine Family and Signaling

The IFN cytokine family comprises type I, type II, and type III IFNs, classified based on the receptor complexes that they interact with. Type I IFNs in humans include multiple IFNα subtypes, IFNβ, IFNε, IFNκ, and IFNω, which all signal through the heterodimeric IFNα receptor (IFNAR) complex [24]. The IFNAR, composed of subunits IFNAR1 and IFNAR2, and its canonical downstream mediators of the JAK and STAT families are widely expressed throughout the body [25]. IFNγ, the only member of type II IFN, is mainly produced by activated immune cells in response to cytokines or antigen-specific stimulation, and signals through the IFNγ receptor (IFNGR) complex [26]. IFNλ1 (IL29), IFNλ2 (IL28A), IFNλ3 (IL28B), and IFNλ4 are type III IFNs that are primarily produced at mucosal sites [27,28]. Functional IFNλ signaling seems to be confined to epithelial cells and immune cells that express both subunits of the IFNλ receptor (IFNLR) complex [29]. Despite signaling through distinct receptor complexes, downstream intracellular signaling pathways and transcriptional responses largely overlap between the types of IFN [25,26,30]. Cellular response to IFN receptor activation is dependent on cell type, context, and timing of the immune response [25].

### 2.2. IFN Induction and Immunomodulatory Functions 

IFNs are named for their capacity to induce an antiviral state in responder cells, thereby interfering with viral replication [9]. In addition, IFNs have a wide range of immunomodulatory functions in orchestrating both innate and adaptive immunity and affecting cellular differentiation, proliferation, and survival [10]. Under homeostatic conditions, type I IFNs are expressed at very low levels, but their expression can be rapidly induced upon signaling of PRRs [31]. Key pathways involved in induction of type I or type III IFNs are PRRs recognizing NAs. These include endosomal Toll-like receptors (TLRs), cytosolic RIG-I like receptors (RLRs), and DNA-sensing receptors (DSRs) [31,32,33]. Plasmacytoid dendritic cells (pDCs), the professional type I IFN-producing cells, are especially equipped to rapidly produce massive amounts of IFNα in response to TLR7 or TLR9 activation [34]. However, virtually every cell type can produce small amounts of type I IFNs and depending on the type and timing of stimulation, different cell types can be the primary source of type I IFNs [35,36,37]. Expression of IFNs is tightly regulated to ensure properly timed robust anti-viral responses, while avoiding excessive tissue damage [31]. Persistent IFN activation, as occurs in systemic autoimmune diseases, drives a detrimental combination of chronic inflammation and immunosuppression contributing to tissue damage and disease progression [11].

### 2.3. Persistent IFN Activation in pSS

Multiple lines of evidence strongly suggest a pathogenic role for IFNs in systemic autoimmune diseases [38]. Anifrolumab, a blocking antibody against the IFNAR, has recently yielded promising results in patients with active systemic lupus erythematosus (SLE) [39,40,41,42]. The so-called type I IFN signature has first been described in SLE when transcriptomic analysis revealed remarkable upregulation of IFN-stimulated gene (ISG) transcripts [43,44]. Expression of ISGs is widely used to assess the activation state of the IFN system. Direct measurement of IFN proteins in patient material is complicated by their diversity and the low circulating levels, although recent development of the single-molecule array (SiMoA) technology has advanced this field [45,46]. 

Transcriptomic analyses on pSS salivary glands have revealed upregulated ISG transcripts in whole salivary gland biopsies [47,48,49,50] and epithelium-enriched fractions [51,52]. Protein expression of type I [14,53,54], type II [55,56], and type III IFNs [57,58] has been observed in salivary glands. Immunostainings indicated pDCs as IFNα-producers, B-, and T-lymphocytes as primary type II-producers and epithelial cells as type I and type III IFN-producing cells in pSS salivary glands [14,53,54,55,57,58,59]. IFN activity in salivary glands has been associated with higher focus score, secretory dysfunction, and higher prevalence of anti-nuclear antibodies and hyperglobulinemia [55,60].

Upregulated ISG expression has also consistently been found in peripheral blood and individual leukocyte subsets from pSS patients [61,62,63,64,65,66]. Serum IFNα levels in pSS are usually below detection limit of conventional enzyme-immunoassays [14]. Application of the SiMoA technology indicated elevated IFNα protein levels in serum compared to controls [67] (and unpublished results). Systemic IFN activity has been primarily linked to serological markers of B cell hyperactivity, complement consumption, and hematological aberrations [55,62,63,67,68]. Autoantibodies against IFNα and IFNω were recently detected in pSS patients [69]. Interestingly, a case with high titer of partially neutralizing anti-IFNα/ω antibodies experienced milder sicca symptoms and minimal focal infiltrates in salivary glands supporting the pathogenic role of IFN in pSS. 

The cellular source of type I IFNs in the circulation remains elusive. Similar to SLE, IFN-producing cells have not been found in the circulation of pSS patients so far, suggesting their migration into tissues [70]. In addition to type I IFNs, also IFNγ appears to contribute to the observed IFN signature in glandular tissue [55] as well as in peripheral blood of a subgroup of patients [68]. In salivary glands, a predominant type II IFN signature is associated with a higher focus score and is commonly found in patients with lymphoma or at high risk for lymphoma [55,60]. The subgroup of patients with additional systemic type II on top of type I IFN involvement have more pronounced serological and hematological manifestations [68]. The interconnection between IFN activation in target tissues and peripheral blood is currently unclear. Research in SLE illustrates that IFN activation does not necessarily co-occur in various tissues and peripheral blood [71,72]. 

## 3. Endosomal TLR Signaling and Involvement in Primary Sjögren’s Syndrome

### 3.1. Endosomal TLR Signaling

TLRs are transmembrane proteins located on the cell surface or endosomal membranes. In humans, 10 types of TLRs have been described, together recognizing a variety of pathogen- and danger- associated molecular patterns [73]. TLR3, TLR7, TLR8, and TLR9 are located on endosomal membranes and respond to various types of NAs delivered to the endosomes through receptor-mediated endocytosis or autophagic delivery [74]. Activating ligands and signaling pathways of endosomal TLRs leading to production of type I IFNs and/or pro-inflammatory cytokines have been summarized by others [7,75,76]. 

Surface receptors such as Fc receptors, complement receptors, B cell receptors, and receptor for advanced glycation end products (RAGE) facilitate efficient endocytosis of TLR ligands [74]. Distinct classes of TLR9-activating CpG oligodeoxynucleotides induce contrasting cytokine responses in pDCs. Class A CpG primary localizes to early endosomes and elicits a type I IFN response through IRF7 activation, while class B CpG preferentially locates to late endosomes resulting in NFκB-mediated production of pro-inflammatory cytokines [77]. This illustrates that differential cytokine responses to TLR activation are at least partially dependent on localization of ligands to distinct endosomal signaling compartments [74].

### 3.2. Endosomal TLRs in pSS

Observations from both mouse and human studies have linked aberrant expression and activation of TLRs to pSS, which has recently been reviewed [78]. Important early work in this field showed the presence of autoantibodies in the circulation of patients with pSS and SLE that have the capacity to form interferogenic RNA-containing immune complexes [14,15]. When mixed with apoptotic or necrotic material, these in vitro formed pSS-derived immune complexes stimulated FcγRIIa (CD32)-dependent and presumably TLR7-mediated IFNα production by pDCs, which could be abrogated by RNase treatment [14,15]. In this study, the interferogenic capacity of serum was associated with the presence of focal infiltrates in salivary glands and several extraglandular manifestations [14]. The specific RNAs causing TLR activation in these experimental conditions have not been elucidated. Later however, anti-Ro60 autoantibodies isolated from SLE sera were found to bind to *Alu* RNA motifs, Y-RNAs, and poly G RNAs [79]. These self-RNAs have the potential to activate NA receptors and elicit cell type specific cytokine responses that are heavily influenced by the (macro)molecular structure and experimental context of RNA delivery [15,79,80,81,82,83]. Similarly, apoptotic material that contains pSS-associated autoantigens and hY RNA elicits TLR-dependent cytokine release in the presence of autoantibodies [83,84]. Additionally, high-mobility group box 1 (HMGB1)-bound DNA, nucleosomes, or immune complexes may activate TLR9 through RAGE-mediated endocytosis [85]. Interestingly, HMGB1 is elevated in serum and released in salivary glands from pSS patients [86]. Given the abundance of cell free DNA in pSS [87,88,89], this pathway could be relevant even in the absence of anti-dsDNA or anti-nucleosome antibodies. Research on type I IFN activation in pSS has been largely focused on TLRs while the other NA receptors have received less attention.

## 4. Monogenic Interferonopathies

Rare monogenic IFN-driven autoinflammatory and autoimmune syndromes provide valuable insights in molecular pathways implicated in type I IFN activation and pathogenic consequences of chronically elevated IFN levels. The majority of mutations causing these inheritable interferonopathies are located in genes related to NA metabolism or NA-sensing receptor signaling [90]. At present, mutations in seven genes have been described to cause Aicardi–Goutières syndrome (AGS), a prototypic autoimmune syndrome [91]. Each of these genes is involved in metabolism of endogenous NAs released during processes such as DNA replication, transcription, and translation. Mutations in the DSR-adaptor protein *TMEM173*/STING induce a prominent type I IFN signature in STING-Associated Vasculopathy with onset in Infancy (SAVI) [91]. In contrast to AGS patients, which often present with a range of autoantibodies at a young age, chronic exposure to IFNs in SAVI may lead to secondary autoimmune features later in life [92]. Therefore, monogenic interferonopathies yield important clues for the interconnection between aberrant IFN activation and autoimmune features.

## 5. Cytosolic IFN-Inducing RNA-Sensing Pathways in Primary Sjögren’s Syndrome

### 5.1. RIG-I Like Receptor Signaling and Other IFN-Inducing RNA-Sensors

The RLRs, an important family of RNA-sensing receptors, play an important role in the defense against viral and intracellular bacterial infections and have been shown to contribute to IFN activation in monogenic interferonopathies and SLE-like syndromes [93]. The RLRs are primarily located in the cytoplasm and expressed by most cell types [8]. The members of this family, RIG-I, MDA5, and LGP2 recognize RNA by coaction of their central helicase domain and carboxy-terminal domain [8]. RIG-I and MDA5 can confer downstream signaling leading through the CARD domain, whereas LGP2, which lacks the CARD domain, is thought to regulate RLR activity. RIG-I mainly recognizes RNAs with a 5′end triphosphate moiety, a biochemical feature that is absent in the majority of cytosolic self-RNAs. RIG-I can also be activated by long dsRNA through a lower affinity binding that is independent of 5′ triphosphate [94]. MDA5 preferentially binds long dsRNAs independent of 5′ triphosphate [95]. Stable ligand binding to RIG-I or MDA5 triggers oligomerization at the CARD domains resulting in formation of helical filaments [96,97]. Recruitment of downstream adaptor protein MAVS and subsequent activation of TBK1, IKKε and transcription factors IRF3, IRF7, and NFκB lead to the production of type I IFNs and other cytokines [8]. 

Multiple other less well characterised RNA-binding proteins and RNA helicases have been described and some of these are proposed to affect IFN signaling [93]. Here we will highlight some elements of protein kinase R (PKR) and RNase L signaling. PKR, encoded by the *EIF2AK2* gene, can be activated by dsRNA and several other stressors [98,99]. PKR is one of the downstream mediators of IFN signaling, but is also involved in the induction of type I IFN expression both as amplifier of IFNβ production and as an essential downstream mediator in MDA5-mediated IFNβ production [100,101]. Catalytic activity of PKR itself may even directly stimulate MAVS-dependent type I IFN production [101]. Upon activation by cytosolic dsRNA, oligoadenylate synthetases (OAS) catalyzes the synthesis of 2′-5′-oligoadenylates, which activates the endoribonuclease RNaseL [102]. In addition to its viral restriction capacity, RNaseL also potentiates immune activation by generating small RNA fragments from viral [103,104] and endogenous RNA [105] that are able to stimulate RLRs or MAVS-dependent NLRP3 inflammasome formation.

### 5.2. Regulation of RNA-Sensing Pathway Signaling

Erroreous activation of RLRs by cellular RNAs is prevented by capping, shielding, and compartmentalization [4,8]. Signaling of RLRs is regulated by posttranslational modifications, protein–protein interactions, non-coding RNAs, and autophagy [4,8]. Sensing of endogenous RNAs has in some instances been shown to potentiate immune responses in the context of infections [105,106,107]. On the other hand, some forms of self-RNA sensing are essential for the prevention of aberrant immune responses. Abundant prevalence of dsRNA-containing circular RNAs prevents PKR activation and subsequent IFN production in homeostatic conditions, but is disturbed in SLE [23]. Dysregulation of RLR signaling or RNA metabolism can lead to activation of RLRs in sterile conditions, as occurs in monogenic interferonopathies and cancer [107]. Anti-tumor effects of some of the classic cancer treatments rely on treatment-induced perturbations in RNA metabolism and induction of type I IFNs [107,108,109,110]. Knowledge on key regulators of RNA metabolism and epigenetic repression of endogenous retroelements acquired by cancer research, may be highly relevant for the field of autoimmunity.

### 5.3. RNA-Sensing Receptors in pSS

Salivary gland epithelium provides an important barrier function as first line defense against infectious agents. In normal salivary gland epithelial cells, RIG-I, MDA5, and PKR proteins are expressed at low levels [64,111,112], but their expression can be rapidly upregulated after stimulation [113]. Downstream adaptor molecule MAVS is highly expressed at protein level in both ductal and acinar epithelium of normal salivary gland [112]. The RLR-MAVS and PKR pathways have been proven functionally active in primary human salivary gland epithelial cells [111,113]. Transcriptomic analyses of pSS-derived salivary gland have repeatedly found upregulation of multiple ISGs involved in cytosolic RNA-sensing pathways or type I IFN signaling [47,48,49,50,114,115,116]. These findings were most pronounced in salivary glands with extensive mononuclear cell infiltrates [50,51,52,114,117]. Prominent staining of RIG-I and MDA5 has primarily been observed in infiltrating immune cells [64], which may be a reflection of localized IFN production.

Immune cells are especially equipped to initiate immune responses to foreign NAs. Most immune cell types express cytosolic RNA-sensing receptors and their downstream signaling molecules at steady state [112]. Considering that the RNA-sensing receptors themselves are IFN-regulated, it is not surprising to see increased gene expression of *IFIH1/MDA5*, *DDX58/RIG-I*, *EIF2AK2/PKR,* and *OAS* in whole blood and specific leukocyte subsets from pSS patients [62,64,66,118,119,120,121]. Studies detecting active signaling and functional properties of these pathways in pSS cells have been very limited so far. Increased levels of phosphorylated TBK1, suggesting activated signaling, have been detected in pDCs from pSS patients with high ISG expression [120]. Together, these expression patterns indicate an immunoactive status of pSS circulating immune cells, which may translate to hyperresponsiveness to NA stimuli and contribute to the ongoing loop of inflammation.

### 5.4. Potential Ligands of Cytosolic RNA-Sensing Pathways in pSS

Various forms of cellular RNA have the potential to induce type I IFNs. Endogenous retroelements, as exemplified by patients with AGS, represent an important potential source of IFN-inducing cellular RNAs (Box A1) [122,123,124,125]. Increased levels of *LINE1* RNA, that were positively correlated to type I IFN expression have been detected in pSS minor salivary glands [59]. In accordance, *LINE1* promotor methylation levels were reduced in pSS salivary glands, which was most pronounced in patients with multiple risk factors for lymphoma [126]. RNA sequencing of SLE peripheral blood mononuclear cells (PBMCs) indicated higher number of *Alu*-derived transcripts compared to control PBMCs [79]. Hitherto, quantification of retroelement-derived transcripts in pSS has been limited to salivary gland tissue.

In addition to endogenous retroelements, other forms of (non-coding) RNAs may induce IFN expression. For example, microRNA (miR)-1248 that regulates calcium signaling has been reported to induce IFNβ by direct RIG-I binding [111]. Overexpression of miR-1248 has been correlated to ISG expression in pSS salivary gland [111]. Mitochondrial RNA leaking into the cytoplasm or released by dying cells could theoretically induce type I IFNs [127], but this mechanism has not been explored in pSS yet. Transfection of total RNA from pSS PBMCs in p125-HEK293 IFNβ reporter cells [128] failed to induce detectable responses, indicating that PBMCs from pSS patients do not contain large quantities of MDA5 or RIG-I stimulating RNAs (unpublished data). 

Viral etiology of pSS has been a longstanding hypothesis and multiple viruses can cause pSS-like sicca symptoms [129,130,131,132,133]. Important immunological and histological differences exist between pSS and infection-associated sicca [130,133]. Even in the absence of active viral infection, traces of viral RNA may remain present after spontaneous resolution or in seronegative occult localized infections [134,135,136,137]. Interestingly, hepatitis D virus (HDV) antigen and viral RNA have been detected in minor salivary glands from a large proportion of HBV-seronegative pSS patients and non-pSS sicca [138]. The cell types infected with HDV have not been identified and it remains unknown whether HDV RNA can be detected systemically. In mice, HDV cannulation induced sialoadenitis and anti-SSA/SSB antibodies [138]. Although HDV induces strong acute type I IFN expression through MDA5, its replication was not affected [139], providing an explanation for its persistence in pSS salivary glands. A risk conferring variant in the gene encoding OAS1 further relates inefficient viral clearance and sustained type IFN production to pSS [140,141]. Functionally, this variant shifts favored splicing sites, producing alternative isoforms with reduced enzymatic activity and/or lower expression levels [140,141,142]. Overall, it cannot be excluded that hidden viral infections may be a source of type I IFN-inducing NAs in some individuals.

In summary, several potential activators of cytosolic RNA-sensing pathways and type I IFNs have been linked to pSS. Studies have been mainly focused on salivary glands and less on circulating immune cells. Therefore, future studies should aim to reproduce these findings, identify the cellular sources of potential ligands, and characterize both tissue and circulating compartments. 

## 6. Cytosolic IFN-Inducing DNA-Sensing Pathways in Primary Sjögren’s Syndrome 

### 6.1. Signaling and Regulation of Cytosolic DNA-Sensing Pathways

Cytosolic dsDNA from both microbial and endogenous origin can be recognized by DSRs mediating protection against pathogens and antitumor immunity [143,144,145]. cGAS is the primary cytosolic DNA sensor [143]. This sensor efficiently recognizes the sugar-backbone of dsDNAs longer than 45 bp with its two main DNA-binding domains in a sequence–independent manner [146,147,148]. Within liquid-phase separated foci, activated cGAS catalyzes the conversion of GTP and ATP to 2′3′cGAMP [149,150]. This second messenger prompts translocation of the adaptor protein STING from the endoplasmic reticulum (ER) into the perinuclear ER intermediate Golgi complex (ERGIC) [151,152]. There, STING recruits and activates downstream kinases IKK and TBK1, which in turn phosphorylate transcription factors IRF3/IRF7 and stimulate type I IFN production [153]. STING can also be activated independent of cGAS by several cyclic dinucleotides [144]. 

Since the discovery of the first cytosolic putative DNA/RNA-sensing receptor DAI/ZBP-1 [154,155], multiple other putative cytosolic DNA sensors have been proposed to induce type I IFN, NFκB-dependent cytokines or inflammasome activation [3,156]. In this context, IFI16 is known to induce ASC-inflammasome formation [157,158,159]. Moreover, IFI16 is an integral part of cGAS-STING signaling pathway ensuring both optimal cGAS-mediated cGAMP production and acting as a crucial mediator for downstream recruitment of TBK1 [160,161]. Other putative DSRs are considered less important than cGAS, but their activation and signaling are still poorly characterized. 

In homeostatic conditions, inappropriate sensing of self-DNA is limited by compartmentalization of cellular DNA and sensors, and metabolization of DNA by endogenous nucleases [144,148]. Signaling of the cGAS-STING pathway is regulated by various epigenetic, transcriptional, and post-translational mechanisms [148]. Defects in these mechanisms can lead to STING-dependent type I IFN production and disease [144,162,163,164]. 

### 6.2. DNA-Sensing Receptors in pSS

Findings from several studies have provided some indications for involvement of cytosolic DNA-sensing pathways in pSS and their potential contribution to type I IFN activation. Protein expression of the DNA sensor cGAS and downstream mediator STING can be readily detected in salivary gland epithelial cells [112,165]. The STING pathway was shown to be functionally active in primary murine salivary gland cells, resulting in the production of type I IFN [165]. In contrast, IFI16 is not constitutively expressed in normal salivary gland epithelium [112,166]. 

Transcriptomic analysis of salivary gland biopsies and epithelium-enriched fractions revealed increased expression levels of *IFI16*, but not *cGAS* or *TMEM173/STING*, in pSS compared to controls [50,51,166,167]. Immunohistochemical staining demonstrated prominent nuclear and cytoplasmic IFI16 expression in pSS salivary gland epithelium [166,168]. In ductal epithelial cells, the cytoplasmic IFI16 was organized in filamentous structures [169], suggesting activation by dsDNA or by spontaneous assembly of high concentrated monomers [169,170,171]. Although not investigated in this study, the activated IFI16 may induce type I IFN production [169]. Notably, the filamentous IFI16 can be effectively bound by anti-IFI16 autoantibodies from pSS patients, which have previously been associated with a more severe disease phenotype [167,169].

Cytoplasmic IFI16 staining has also been shown for infiltrating immune cells [166], but the macromolecular organization of IFI16 has not been reported. Transcript levels of *IFI16*, but not *TMEM173/STING*, are elevated in peripheral blood [62,119,121] and circulating pDCs [64,66] from pSS patients. Although pDCs are primarily known for their responsiveness to TLR7 and TLR9 ligands, they also possess a functional cGAS-STING pathway. Stimulation of this pathway in pDCs inhibits TLR9 activity [172]. In this context, it is worth noting that pDCs from both pSS and SLE patients tend to produce less type I IFN in response to TLR9 stimulation [66,173]. Interestingly, increased levels of cGAMP, indicative for active STING signaling, have been reported in PBMCs from SLE patients [20,174]. Indications for activation of cGAS-STING pathway in pSS are still lacking.

Summarizing, current literature suggests activated IFI16 signaling in pSS salivary glands which may potentially contribute to type I IFN production. 

### 6.3. Potential Ligands of Cytosolic DNA-Sensing Pathways in pSS

Cytoplasmic deposition of genomic or mitochondrial DNA caused by perturbations in DNA metabolism may elicit DNA-sensing pathway activation and type I IFN production. pSS-derived salivary gland epithelial cell lines and salivary gland biopsies contain cytosolic dsDNA depositions that have been linked to reduced DNase I activity and AIM2 inflammasome activation [175]. The authors did not detect strong expression of ISGs and hence consider type I IFN induction by dsDNA depositions in these salivary gland epithelial cell lines unlikely [117]. Similar cytoplasmic DNA depositions have been identified in salivary gland infiltrating macrophages in pSS that show signs of NLRP3 inflammasome activation and pyroptotic death [89]. In accordance with this inflammatory type of cell death, dsDNA strands were observed in the extracellular space around ductal structures. 

The ATP-gating ion channel and pore-forming P2X7 receptor (P2X7R) that regulates inflammasome activation has recently also been implicated in ATP-gated cGAMP transport from the extracellular space mediating STING-dependent type I IFN production [176,177]. P2X7R is abundantly present on immune cells and salivary gland epithelium [112,178]. A genetic variant in *P2X7R* has been linked to seropositive pSS in individuals not carrying *HLA-DR3* risk alleles [179]. Additionally, P2X7R is expressed at higher levels in salivary glands of pSS and its expression is correlated to focus score and lymphoma development [180,181]. These data indicate that P2X7R might play a role in autoinflammation in pSS salivary glands by impacting various innate immune functions. 

Reduced DNAse II activity and increased amounts of short-fragmented cytosolic DNA have been reported in PBMCs from pSS patients, most notably in patients with a high-risk phenotype for lymphoma development [89]. DNase II is highly expressed in lysosomes of macrophages and degrades DNA internalized by endocytosis or through phagocytosis of apoptotic bodies [182]. Mice lacking DNase II develop an autoimmune-like phenotype and anti-nuclear antibodies [164,183]. In the absence of DNase II, insufficient elimination of DNA from the lysosomes results in leakage of undigested DNA into the cytoplasm [89] inducing STING-dependent type I IFN production [184].

Excessive DNA damage may disturb the balance between supply and digestion of DNA fragments causing cytosolic DNA accumulation and thereby providing ligands for the cytosolic DSRs. Several studies describe excessive DNA damage in pSS salivary glands, specifically in the lymphoepithelial lesions [175,185]. The endonuclease TREX1 is one of the crucial mediators in cytosolic DNA degradation. In pSS, several genetic variants in *TREX1* have been described [186]. Interestingly, one *TREX1*-variant has been negatively associated with pSS-lymphoma development and positively associated with elevated ISGs in minor salivary glands [187].

In addition to genomic DNA, mitochondrial DNA released into the cytoplasm is able to activate DNA-sensing pathways. Leakage of mitochondrial DNA from damaged mitochondria resulting from excessive IFNα signaling and impaired autophagy has recently been shown to induce STING signaling in SLE monocytes [21]. In pSS, disturbances in autophagy [188,189,190] have not been investigated in the context of type I IFN production.

In conclusion, impaired DNA clearance, leakage of DNA from intracellular compartments, and excessive DNA damage contribute to accumulation of genomic DNA in pSS. How these extracellular and cytosolic DNA depositions relate to type I IFN activation in pSS patients remains to be elucidated.

## 7. Crosstalk between Nucleic Acid-Sensing Pathways

The NA-induced signaling pathways can hardly be considered in isolation as significant crosstalk between these pathways occur at multiple levels [191]. First, expression levels of cytosolic and endosomal NA-sensing receptors are often coregulated [191]. Second, distinct NA-sensing receptor families may share part of the downstream signaling components [192]. Third, modification of NAs may result in activation of multiple NA sensors in parallel. For example, RNAs can be reverse transcribed into DNA creating ligands for the DNA-sensing pathway. RNA polymerase III is able to transcribe 5′-triphosphate-containing RNAs from dsDNA, creating ligands for RIG-I [193,194]. Fourth, NA signaling pathways also negatively regulate each other, which is illustrated by the seemingly contradictory roles of STING in autoimmunity. Knockout of *STING* has been reported to resolve autoimmunity in some animal autoimmune models [162,163,164], while exacerbating autoimmunity in other models [195]. The latter observations could at least in part be explained by the negative regulatory role of STING on TLR7 and/or TLR9 signaling in certain immune cells, which is potentially mediated through SOCS1/3 [172,195]. Similar mechanisms may explain some of the conflicting reports on other immune sensors in autoimmune models as well. Recently, SID1 transmembrane family member 2 (SIDT2) has been identified to transport dsRNA from the endolysosomal compartment to the cytoplasm where it can stimulate the cytosolic NA receptors [196], integrating NA-sensing pathways from different cellular compartments. 

## 8. Extracellular Nucleic Acids: Potential Ligands for Cytosolic Nucleic Acids Sensors? 

Defects in regulation of apoptosis or clearance of cellular debris can cause rare forms of monogenic SLE or increase the risk for SLE development [90,197]. Observations from pSS patients and mouse models suggest abnormal regulation of apoptosis, which has been considered a key mechanism for exposure of autoantigens to the immune system [83,198,199]. 

If not cleared effectively, apoptotic cells may undergo secondary necrosis and leak their intracellular components causing immune activation [200]. Multiple studies indicated impaired clearance of apoptotic cells in pSS patients [201,202,203] and increased levels of circulating nucleosomes and cell free DNA [87,88]. These levels were inversely correlated to the activity of serum DNase I, which digests excessive extracellular DNA [88,89]. Autoantibodies against DNase I were described in almost half of the studied pSS sera [204]. Although not experimentally proven yet, analogous associations of several serological markers with both anti-DNAse I autoantibodies [204] and plasma cell free DNA [87] suggest neutralizing ability of these antibodies. Similar autoantibodies in SLE have already been shown to inhibit the DNase I enzymatic activity [205].

Opsonization of insufficiently degraded apoptotic cell remnants by pSS-derived autoantibodies drive efficient phagocytosis [88]. Even intact apoptotic bodies are potentially immunogenic as illustrated by the secretion of pro-inflammatory cytokines by pDCs following internalization of these extracellular vesicles [83]. Apoptosis-derived membrane vesicles that are present in serum from SLE patients have been shown to induce cGAS-STING–driven ISG expression [174]. The pSS sera that were tested in this study did not have this STING-mediated IFN-inducing capacity. 

Recently, a new type of highly immunogenic extracellular vesicle has been described that is produced by various cell types during apoptosis [206]. These apoptotic exosome-like vesicles contain non-coding RNAs with molecular structures allowing the recognition by RLRs and endosomal TLRs [95]. The same immunogenicity and type of cargo was described for exosomes released from various cancer cell types that were shown to stimulate IFN production through TLR3 and RLRs [207,208]. Considering that these exosome-like vesicles seem to be produced by damaged or abnormal cells, these vesicles may also be released in autoimmune diseases characterized by excessive tissue damage.

In conclusion, dysregulation of apoptosis and impaired clearance of cellular debris in pSS promote exposure of autoantigens and NAs to the immune system. Depending on macromolecular presentation, efficiency of endolysosomal degradation, and cell type, these internalized NAs may activate endosomal TLRs and/or cytosolic NA sensors.

## 9. Conclusions and Future Directions

**Figure 1:**
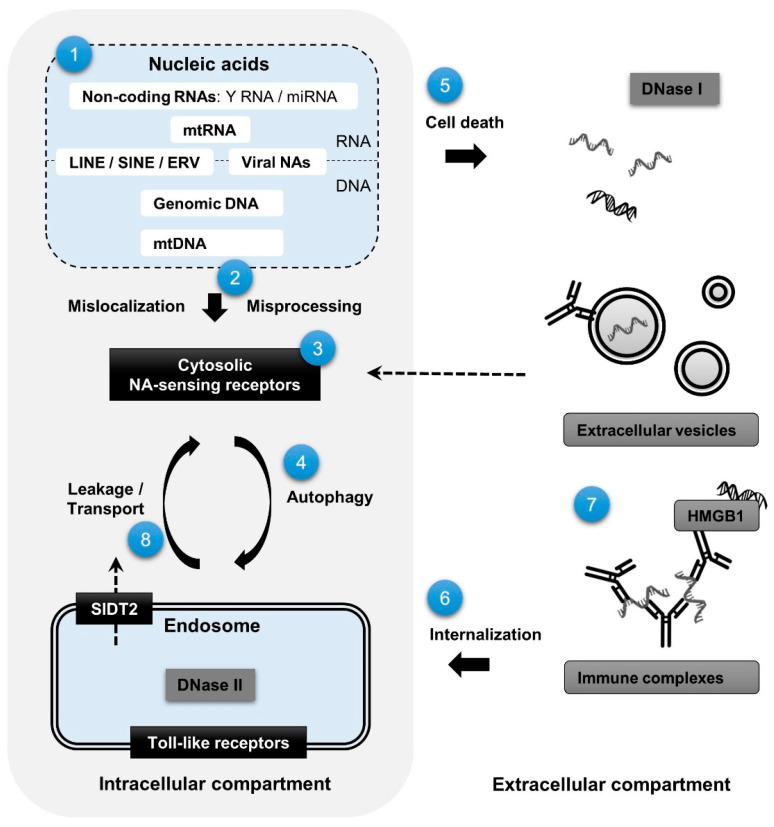
Graphical overview of mechanisms potentially contributing to activation of intracellular nucleic acid-sensing pathways and type I IFN production in primary Sjögren’s syndrome. Intracellular nucleic acids (NAs) from viral or endogenous origin (**1**) may, as a result of abundant expression, misprocessing or mislocalization (**2**), be sensed by cytosolic NA receptors (**3**), be delivered to the endolysomal compartment through autophagy (**4**), or be released into the extracellular space through cell death or active secretion (**5**). If not cleared sufficiently by nucleases or efferocytosis, internalization of extracellular NAs by immune cells (**6**) may lead to activation of endosomal Toll-like receptors. Extracellular NAs can exist in various macromolecular complexes (**7**) that impact their immunogenicity, the efficiency and mechanism of internalization, and cellular response. NAs confined to the endolysosomal compartment may escape into the cytoplasm via specialized transporters or as a result of inefficient digestion and loss of endosomal integrity (**8**).

Knowledge from multiple disciplines has advanced our understanding of potential driving mechanisms of type I IFN activation in systemic autoimmune diseases. NAs when highly abundant, incorrectly processed, or mislocalized may activate cytosolic PRRs or enter the extracellular compartment from which they can be internalized by immune cells and activate endosomal PRRs (Figure 1). Multiple observations support a potential role for both intra- and extracellular NAs in type I IFN activation in pSS. Dysregulation of endolysosomal digestion and autophagic trafficking under inflammatory pressure may integrate signaling via the endosomal and cytosolic NA-sensing pathways in pSS. Interestingly, hydroxychloroquine affects both endolysosomal maturation and autophagic flux. Therefore, it is highly relevant to study the impact of regularly used medications on NA processing and the various sensing pathways. Future research should identify the cellular sources of IFN-inducing NAs and study negative regulatory mechanisms. Advanced techniques such as single cell sequencing and spatial transcriptomics will elucidate cell specific alterations in relation to their localization in tissue. Detailed characterization of activated NA-sensing pathways in individual patients may shed light on clinical heterogeneity and has important implications for treatment of systemic autoimmune diseases.

## Data Availability

No new data were created or analyzed in this study. Data sharing is not applicable to this article.

## Appendix A

Box A1 Transposable elements. A large proportion of the human genome is composed of transposable elements which can integrate in new locations in genome. These transposable elements can be classified into those with long terminal repeats (LTRs), the endogenous retroviruses (ERVs), and those without LTRs, which include the short interspersed nuclear elements (SINE) and long interspersed nuclear elements (LINE) [209]. Retrotransposon activity occasionally leads to disease when causing frame shifts, splicing defects or deletions and seems to contribute to genetic diversity related to disease risk [210]. *LINE1* is an autonomous transposon that is currently active in human genomes [211,212]. *LINE1* is transcribed by RNA polymerase II and encodes a RNA-binding protein, a reverse transcriptase and an endonuclease [209]. A large proportion of SINE are *Alu* retroelements that require LINE1 activity for transposition [213]. *Alu* RNAs are heavily involved in regulation of transcription, splicing, RNA stability, and translation [214]. The majority of expressed *Alu* RNAs are located within polymerase II-transcribed mRNAs [214]. *Alus* located outside coding regions can be transcribed by polymerase III, a process that occurs at low levels in healthy cells, but can be induced by various stressors [213,214,215,216]. Endogenous retroelement-derived RNA transcripts contain biochemical structures or form secondary and tertiary structures that—in case of improper processing—can activate TLRs, RLRs and PKR and induce type I IFN production [59,106,123,124,125,213,217,218,219]. Reverse transcribed retroelemental cDNA and DNA fragments from transposon-induced DNA damage are potential triggers of the cGAS-STING pathway [59,123].
